# Urinary output and fractional excretion of sodium and urea as indicators of transient versus intrinsic acute kidney injury during early sepsis

**DOI:** 10.1186/cc13057

**Published:** 2013-10-13

**Authors:** Jill Vanmassenhove, Griet Glorieux, Eric Hoste, Annemieke Dhondt, Raymond Vanholder, Wim Van Biesen

**Affiliations:** 1 Renal Division, Ghent University Hospital, Gent, Belgium; 2 Intensive Care Unit, Ghent University Hospital, Gent, Belgium; 3 Senior Clinical Investigator – Research Foundation Flanders, Gent, Belgium

## Abstract

**Introduction:**

The pathophysiology of acute kidney injury (AKI) in sepsis is ill defined. We investigated parameters associated with low glomerular filtration, and their predictive value to discriminate transient from intrinsic septic AKI.

**Methods:**

In 107 sepsis patients, AKI was defined by the Risk, Injury, Failure, Loss of Kidney Function, End-stage renal disease (RIFLE) urinary output or serum creatinine criterion, or both. Transient AKI (TAKI) versus intrinsic AKI was defined as RIFLE R, I, or F on the first day evolving to no AKI or not, respectively, over the following 5 days. Fractional excretion of sodium (FENa), urea (FEUrea), and NGAL (FENGAL) at admission (d0t0), 4 (d0t4), and 24 hours (d1) was determined.

**Results:**

Including versus not including the urinary-output criterion of RIFLE increased AKI from 43% to 64.5%. Median uNGAL levels and FENGAL were lower in no AKI versus transient AKI when AKI was defined based on creatinine (*P* = 0.002 and *P* = 0.04, respectively), but not when based on urinary output (*P* = 0.9 and *P* = 0.49, respectively). FENa < 1% and FEUrea <35% was present in 77.3% and 63.2% of patients. Urinary NGAL was higher (*P* < 0.001) in those with high versus low fractional sodium excretion, but this was only in patients with transient or intrinsic AKI (*P* < 0.001 in subgroups), and not in patients without AKI. The negative predictive value for either intrinsic AKI or not restoring diuresis in patients with FENa > 0.36% and FEUrea > 31.5% was 92% and 94.5% respectively.

**Conclusions:**

A low FENa and FEUrea is highly prevalent in the first hours of sepsis. In sepsis, oliguria is an earlier sign of impending AKI than increase in serum creatinine. A combination of a high FENa and a low FEUrea is associated with intrinsic AKI, whereas a combined high FENa and FEUrea is strongly predictive of transient AKI.

## Introduction

Controversy exists about the role of low glomerular filtration pressure in the pathogenesis of septic acute kidney injury (AKI) [1-3]. Tubular damage due to inflammatory cascades is also proposed as a potential underlying pathophysiological mechanism [4-7]. Recently, it was demonstrated that transient azotemia can be more than a physiologic response to low glomerular filtration pressure, as it can also be associated with transient low-grade tubular injury, as defined by the presence of neutrophil gelatinase-associated lipocalin (NGAL) [8] or liver fatty acid binding protein (LFABP) [9]. Whereas these findings enhance our understanding of the pathophysiology of AKI, they are not always very helpful in clinical practice, in which the most important dilemma is to differentiate those cases in which restoration of glomerular filtration pressure can potentially still reverse AKI from those in which it will lead only to fluid overload. This is important, as increasing evidence links blind fluid loading to higher mortality [10,11].

Although most definitions of AKI are based on both an increase in serum creatinine and a decrease in urinary output, the latter criterion has been neglected in many studies [12-15]. Recent work has demonstrated that urinary output is important, as it is associated with morbidity and mortality [16]. A low urinary output can be used as an early-warning parameter of developing AKI, maybe even before tubular damage arises.

We hypothesized that the combined interpretation of a reduced urinary output, and a low fractional excretion of sodium and urea, and especially the evolution of these parameters, is an early indicator of incipient AKI in sepsis patients, when it is still reversible if glomerular filtration pressure is restored. We also hypothesized that a high fractional sodium excretion can be an indicator of active sodium secretion by damaged tubular cells and reduced proximal tubular reabsorption, whereby extent of tubular damage was assessed by (fractional excretion of) urinary neutrophil gelatinase-associated lipocalin (NGAL).

## Materials and methods

The study was approved by the ethical committee of the Ghent University Hospital. Written informed consent was obtained from the patients or their next of kin.

In total, 107 consecutive patients with sepsis admitted to the intensive care unit (ICU) of a tertiary university hospital were included between 12/01/2010 and 5/09/2010. Sepsis, severe sepsis, and septic shock were defined according to the American College of Chest Physicians/Society of Critical Care Medicine Consensus Conference guidelines [17]. Exclusion criteria were (a) a history of liver and/or kidney transplantation, (b) ICU stay less than 24 hours, (c) patients treated with chronic hemodialysis, and (d) age younger than 17 years.

Acute kidney injury (AKI) was defined according to RIFLE classification (Risk, Injury, Failure, Loss of kidney function, and End-stage renal disease) [13]. Baseline creatinine was based on the most recent value before admission or was calculated if the latter was not available [13]. The urinary-output criterion was based on 6-hour blocks, as described by Macedo *et al*. [16]. As we wanted to assess the impact of the creatinine and the urine output criteria separately, we defined AKI based on both criteria (AKIboth) together, versus on the urinary output criterion only (AKIuo), or the serum creatinine criterion alone (AKIc). Transient AKI (TAKI) was defined as RIFLE R, I, or F on day 1 that improved to “no AKI” in the following 5 days, whereas “intrinsic AKI” was defined as patients with RIFLE R, I, or F who did not evolve to “no AKI” in the following 5 days.

Urine and blood samples were collected at the moment of admission (time point d0t0), and also 4 hours later (time point d0t4) and 24 hours later (time point d1), to assess the evolution of the parameters. Blood samples were centrifuged within 20 minutes after collection at 1,500 *g* for 10 minutes, and serum was aliquoted and stored at −80°C for later batch analysis. Urine was collected in a sterile way and centrifuged at 500 *g* for 10 minutes; urine samples were aliquoted and stored at −80°C for later batch analysis.

Serum and urinary neutrophil gelatinase-associated lipocalin (NGAL) were measured by using an ELISA kit (Gentofte (Denmark) ^R^Bioporto Diagnostics).

Fractional excretion of sodium, urea, and NGAL was calculated according to the formula (U_x_ × Screa)/(Ucrea x S_x_) × 100 with x = either sodium, urea, or NGAL. Patients were classified according to FEUrea and FENa quartiles. We also defined FENA and FEUrea dichotomously as “high” (two upper quartiles) and “low” (two lower quartiles).

### Statistical analysis

Results are reported as medians and interquartile ranges (IQRs) for continuous variables, unless otherwise specified. Discrete variables are reported as numbers and/or percentages. All statistical analyses were performed by using SPSS 19. All consecutive patients were included, irrespective of their course or duration of stay at the ICU.

Continuous variables were compared between groups by using the Mann–Whitney *U* test (two groups) or the Kruskal-Wallis (more than two groups), as appropriate, by using the nonparametric section of SPSS 19. When the overall *P* value was <0.05, *post hoc* analysis between different pairs was performed by using Mann–Whitney *U* tests, taking into account correction for multiple testing.

Dichotomous variables in groups were compared by using χ^2^ analysis. Positive and negative predictive values were calculated for high and low fractional excretion of sodium and urea, whereby “high” and “low” where based on the median value. We performed Receiver Operating Characteristic (RoC) curves to assess the performance of fractional excretion of sodium and urea to predict transient or intrinsic AKI.

Performance of the combined interpretation of fractional excretion of sodium and urea was evaluated by calculating positive and negative predictive power of the different combinations for restoration of diuresis and for development of intrinsic AKI.

## Results

**Figure 1:**
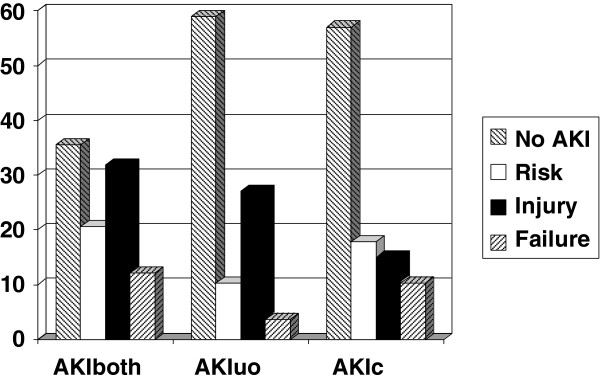
**Distribution of RIFLE class based on a single or both criteria.** Based on the urinary and the creatinine criteria together (AKIboth), 35.5% of patients had no AKI, versus 20.6%, 31.8%, and 12.1% with RIFLE R, I, and F. Based on the urinary output criterion only (AKIuo), 58.9% patients were classified as having no-AKI, versus 10.3%, 27.1%, and 3.7% as RIFLE-R, I, or F, respectively. Based on the creatinine criterion only (AKIc), 57% had no AKI, versus 17.8%, 15%, and 10.3% classified as RIFLE R, I, and F (*P* < 0.03).

In total, 107 consecutive patients were included. Demographic and clinical data are shown separately for AKI versus no-AKI patients, as defined by RIFLE on the first day of admission (Table 1). Demographic and clinical data for no-AKI versus transient AKI versus intrinsic AKI are shown in Table 2. Based on both RIFLE criteria combined (AKIboth), 35.5% of patients had no AKI, and 20.6%, 31.8%, and 12.1% had RIFLE R, I, and F. When AKI was defined based on urinary output alone (AKIuo), 58.9% of patients were classified as having no AKI, and 10.3%, 27.1%, and 3.7% had RIFLE-R, I, or F, respectively. When defining AKI based on the creatinine criterion alone (AKIc), 57% had no AKI, and 17.8%, 15%, and 10.3% were classified as RIFLE R, I, and F (Figure 1), so omitting the urinary-output criterion leads to underdiagnosis of AKI.

**Table 1:** Clinical and demographic characteristics of the study cohort comparing patients without versus those with AKI, as defined by RIFLE on the first day of admission

	**No AKI (*n *** **= 38)**	**AKI (*n *** **= 69)**	***P *** **value**
Gender male (%)	57.9	55.1	0.78
Age (years, mean/SD)	57.5 (16.0)	62.7 (14.2)	0.09
CKD on admission (MDRD <60 ml/min/1.73 m^2^) (%)	10.5	8.7	0.76
APACHE II score on the first day of admission	21 (10)	23 (9)	0.19
Positive fluid balance first 24 hours (mean/SD)	2.5 (2.1)	4.0 (2.3)	0.002
Use of diuretics on the first day of admission (%)	18.4	17.4	0.89
RRT need during ICU stay (%)	5.3	17.4	0.08
Vasopressor use (%)	42.1	68.1	0.009
Total dose of noradrenaline first 24 hours (μg/kg/min)	0.15 (0.25)	0.13 (0.20)	0.83
Maximum dose of noradrenaline during first 24 hours (μg/kg/min)	0.32 (0.44)	0.34 (0.51)	0.83
Need for ventilation during ICU stay (%)	47.4	59.4	0.23
LOS (days)	5 (9)	6 (12)	0.82
ICU mortality (%)	21.1	27.5	0.46
Mortality at 90 days (%)	26.3	36.2	0.30

**Table 2:** Clinical and demographic characteristics of the study cohort comparing patients with no AKI versus transient AKI versus intrinsic AKI

	**No AKI (*n *** **= 28)**	**Transient AKI (*n *** **= 57)**	**Intrinsic AKI (*n *** **= 22)**	***P *** **value**
Gender male (%)	51.7	54.4	59.1	0.93
Age (years, mean/SD)	55.4 (17.3)	62.6 (13.3)	63.1 (14.9)	0.08
CKD on admission (MDRD <60 ml/min/1.73 m^2^) (%)	10.7	8.8	9.1	0.96
APACHE II score on the first day of admission	21 (10)	22 (8)	24.5 (9)	0.08
Positive fluid balance first 24 hours (mean/SD)	2.2 (1.8)	3.3 (1.9)	5.4 (2.7)	<0.001
Use of diuretics on the first day of admission (%)	7.1	19.3	27.3	0.16
RRT need during ICU stay (%)	0	1.8	59.1	<0.001
Vasopressor use (%)	32.1	61.4	86.4	<0.001
Total dose of noradrenaline first 24 hours (μg/kg/min)	0.13 (0.22)	0.12 (0.19)	0.16 (0.28)	0.65
Maximum dose of noradrenaline during first 24 hours (μg/kg/min)	0.28 (0.34)	0.32 (0.47)	0.49 (0.68)	0.66
Need for ventilation during ICU stay (%)	39.3	50.9	86.4	0.003
LOS (days)	5 (6)	5 (10)	38 (32)	0.014
ICU mortality (%)	21.4	15.8	54.2	0.002
Mortality at 90 days (%)	28.6	24.6	59.1	0.012

Upper cut-off values for the FENa quartiles based on the values of d0t0 were 0.15%, 0.36%, and 0.95%. At time points d0t0, d0t4, and d1, 77.3%, 74.5%, and 71.1% of patients had a fractional excretion of sodium (FENa) <1%, the currently used cut-off value.

Upper cut-off values for the FEUrea quartiles were 21%, 31.5%, and 42.0%. At time points d0t0, d0t4, and d1, 63.2%, 50.9%, and 41.3% of patients had a fractional excretion of urea (FEUrea) <35%.

There was a stepwise increase in prevalence of AKI based on urinary output, with quartiles of decreasing FENa and FEUrea at d0t0 (*P* = 0.05 and *P* = 0.01) and d0t4 (*P* < 0.001 for both), but not at d1 for FENa (*P* = 0.18). No such association was noted for AKI based on creatinine (Table 3).

**Table 3:** Prevalence of AKIuo and AKIc (percentage of all patients classified in that category) in different FENa and FEUrea quartiles

FENa quartiles	AKIuo d0t0 *P* = 0.05	AKIuo d0t4 *P* < 0.001	AKIuo d1 *P* = 0.18
<0.15	57.7	72.4	53.3
0.15-0.36	48.1	34.6	46.7
0.36-0.95	33.3	39.1	33.3
>0.95	23.1	14.3	28.1
FEUrea quartiles	AKIuo d0t0 *P* = 0.01	AKIuo d0t4 *P* < 0.001	AKIuo d1 *P* = 0.01
<21	59.3	77.8	83.3
21-31.5	48.1	55.5	38.1
31.5-42	37.5	22.2	30
>42	17.9	23.5	34.1
FENa quartiles	AKIc d0t0 *P* = 0.76	AKIc d0t4 *P* = 0.66	AKIc d1 *P* = 0.09
<0.15	34.6	37.9	33.3
0.15-0.36	40.7	46.2	40
0.36-0.95	48.1	34.8	29.6
>0.95	46.2	50	59.4
FEUrea quartiles	AKIc d0t0 *P* = 0.06	AKIc d0t4 *P* = 0.17	AKIc d1 *P* = 0.72
<21	55.6	61.1	33.3
21-31.5	51.9	44.4	33.3
31.5-42	20.8	44.4	46.7
>42	39.3	29.4	43.9

*P* values represent χ^2^ over the quartiles.

**Figure 2:**
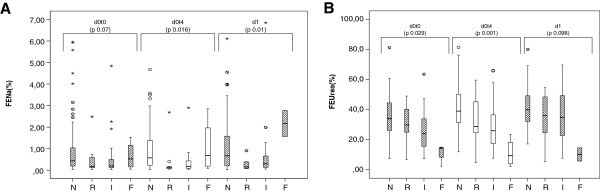
**FENa(%) and FEUrea(%) at the different time points in AKIuo. (A)** U-shaped form of FENa over the different AKIuo classes (N, no AKI; R, Risk; I, Injury; and F, Failure; (*P* = 0.07, 0.016, and 0.01 at d0t0, d0t4, and d1, respectively). **(B)** Decreasing trend in FEUrea(%) across AKIuo classes at the different time points with persistently low FEUrea(%) in RIFLE F class at the three time points (*P* = 0.029; *P* = 0.001; and *P* = 0.098).

The values for FENa and FEUrea in the different AKI stages based on urinary output are illustrated in Figure 2A and B, respectively. FENa was <1% in the majority of patients at all time points, except in patients classified as F at timepoint d1, where the median was 2.16%. A U-shaped pattern of FENa was seen over the different AKI stages based on urinary output (*P* = 0.07, 0.016, and 0.01 at d0t0, d0t4, and d1, respectively) (Figure 2A). There was a decreasing trend in FEUrea at d0t0,d0t4, and d1 across different AKI stages based on urinary output (Figure 2B). Patients with stage F AKIuo had a persistent FEUrea <20% at the three time points (Figure 2B).

To analyze the discriminative power of fractional excretion of sodium and urea and of urinary NGAL to discriminate transient and intrinsic AKI, we performed receiver operating characteristic curves, yielding areas under the curves of 0.59, 0.36, and 0.67, respectively, pointing out that none of these parameters had sufficient power to discriminate intrinsic from transient AKI.

We wanted to assess the predictive value of a combined interpretation of fractional excretion of sodium and urea for the evolution of diuresis and AKI. Therefore, a categoric representation of percentages of patients with no AKI, transient AKI, and intrinsic AKI in different subgroups of high and low fractional excretion of sodium and urea at 4 hours is cross-tabulated in Table 4 (A, based on urinary output: *P* < 0.001; B, based on creatinine: p = 0.01; C, based on both urinary output and creatinine: *P* < 0.001). The negative predictive value for intrinsic AKI for patients with both high fractional excretion of sodium and urea was 92.0%; the negative predictive value that urinary output would not restore in patients with this combination was 94.9%. In contrast, the positive predictive value of a high fractional sodium excretion in combination with a low fractional urea excretion for persisting oliguria was 54%. Thus, a high fractional sodium and a urea excretion are nearly always associated with transient AKI and restoration of diuresis, whereas in patients with a high fractional sodium excretion in combination with a low fractional urea excretion, there is substantial risk for persisting oliguria. The same evaluation for the same parameters at admission showed the same trend (data not shown).

**Table 4:** Cross-tabulation of FENa <0.36%/FEurea <31.5% versus FENa >0.36%/FEurea <31.5% versus FENa <0 36%/FEurea >31.5% versus FENa >0.36%/FEurea >31.5% in AKIuo (A), AKIc (B), and AKIboth (C)

A. AKIuo (% within FENa/FEUrea category)					
	FENa < 0.36%	FENa > 0.36%	FENa < 0.36%	FENa > 0.36%	Total
	FEUrea < 31.5%	FEUrea < 31.5%	FEUrea > 31.5%	FEUrea > 31.5%	
No AKI	18.8	7.7	43.5	71.8	42.1
Transient AKI	62.5	38.5	52.2	23.1	43.0
Intrinsic AKI	18.8	53.8	4.3	5.1	15.0
B. AKIc (% within FENa/FEUrea category)					
No AKI	46.9	30.8	65.2	56.4	52.3
Transient AKI	34.4	15.4	21.7	35.9	29.9
Intrinsic AKI	18.8	53.8	13.0	7.7	17.8
C. AKIboth (% within FENa/FEUrea category)					
No AKI	9.4	0.0	30.4	46.2	26.2
Transient AKI	68.8	38.5	52.2	46.2	53.3
Intrinsic AKI	21.9	61.5	17.4	7.7	20.6

AKIuo, AKIc, AKIboth: Acute kidney injury based on urinary output (uo), serum creatinine (c), or both criteria (both).

A: *P* < 0.001, B: *P* = 0.01 and C: *P* < 0.001.

**Figure 3:**
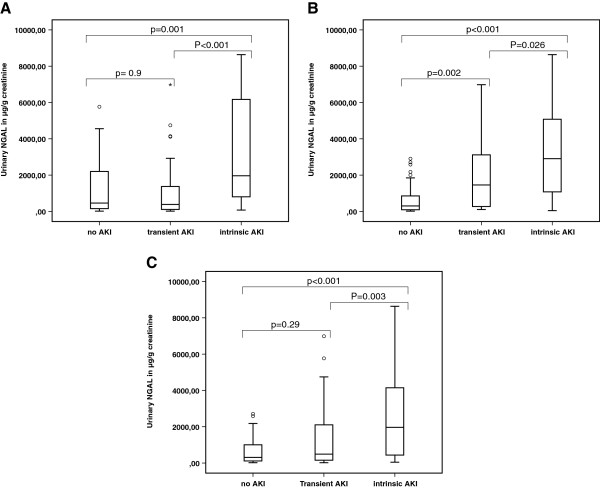
**uNGAL in no-AKI, transient AKI, and intrinsic AKI based on either one or both RIFLE criteria. (A)** AKI based on the urinary output criterion (AKIuo). No significant difference (*P* = 0.9) occurred in uNGAL levels between transient AKI and no-AKI. **(B)** AKI based on the serum creatinine criterion (AKIc). There is a significant difference (*P* = 0.002) in uNGAL levels between transient AKI and no-AKI. **(C)** AKI based on both criteria (AKIboth). As in A, there is no significant difference between transient AKI and no-AKI (*P* = 0.29).

Urinary NGAL values at 4 hours in patients with no AKI, transient AKI, and intrinsic AKI are represented in Figure 3, and this separately for AKI defined on urinary output (Figure 3A), on creatinine (Figure 3B), or both criteria (Figure 3C) (*P* value overall < 0.001). In *post hoc* analysis, a difference in median NGAL levels occurred between no AKI and transient AKI when AKI was defined based on creatinine (*P* = 0.002) whereas there was no difference when it was based on urinary output (*P* = 0.9). A similar gradual increase occurred in the fractional excretion of NGAL in no-AKI versus transient AKI (*P* = 0.04) versus intrinsic AKI when AKI was defined based on the creatinine criterion but again, there was no significant difference between no-AKI and transient AKI (*P* = 0.49) when AKI was defined according to the diuresis criterion only (graphic illustration similar to Figure 3; not shown).

**Figure 4:**
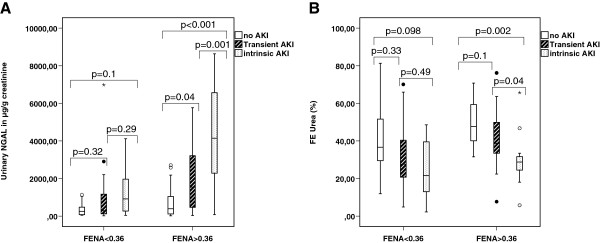
**uNGAL (μg/g creatinine) and FEUrea (%) according to FENa (either <0.36% or >0.36%) in patients with noAKI, transient AKI, and intrinsic AKI. (A)** For patients with FENa <0.36%, uNGAL(μg/g creatinine) levels were not significantly different between no-AKI, transient AKI, and intrinsic AKI. For patients with FENa >0.36%, uNGAL levels were significantly different between transient AKI and intrinsic AKI (*P* = 0.001). **(B)** For patients with FENa <0.36%, there was no significant difference in fractional excretion of urea between no-AKI, transient AKI, and intrinsic AKI. For patients with FENa >0.36%, there was a significant difference between patients with transient AKI and intrinsic AKI (*P* = 0.04).

Urinary NGAL levels and fractional excretion of urea in patients with no AKI versus transient versus intrinsic AKI, and separately for those with a fractional sodium excretion below or above 0.36% are depicted in Figure 4A and B, respectively. Median urinary NGAL was higher in the overall group in those with high (>0.36%) versus low (<0.36%) fractional sodium excretion (median, 1,005 versus 314 μg/g creatinine, respectively; *P* 0.025). In those with high fractional sodium excretion (>0.36%), urinary NGAL was higher in those with intrinsic AKI versus in those with transient AKI (median, 4,146 versus 1,544 μg/g creatinine; *P* = 0.001). In those with a low fractional sodium excretion <0.36%, the difference was not significant (917 versus 286 μg/g creatinine; *P* = 0.29) (Figure 4A). Median fractional excretion of urea was higher (39.6% versus 28.6%; *P* = 0.02) in the overall group in those with high (>0.36%) versus low (<0.36%) fractional sodium excretion. In those with high fractional sodium excretion (>0.36%), fractional urea excretion was lower in those with intrinsic AKI versus those with transient AKI (28.8% versus 38,7%; *P* = 0.04). An RoC analysis of fractional urea excretion in patients with a fractional sodium excretion >0.36% yielded an AUC of 0.76 for discrimination between transient and intrinsic AKI. In those with a low fractional sodium excretion <0.36%, the difference was not significant (21.6% versus 27.6%; *P* = 0.49) (Figure 4B).

The median volume of HES administered in no-AKI versus transient AKI based on urinary output was 0.5 L versus 0.53 L (*P* = 0.15). Based on the creatinine criterion, this was 0.5 L and 0.5 L in no-AKI versus transient AKI (*P* = 0.64).

## Discussion

When urinary output was used on top of serum creatinine in this cohort of sepsis patients to define AKI, more cases of AKI were detected as compared with when AKI was based on only the creatinine criterion. More important, transient AKI based on the RIFLE urinary output criterion was not associated with tubular damage as assessed by urinary NGAL, whereas tubular damage was found when diagnosis of transient AKI was based on the RIFLE creatinine criterion. Our data indicate that in patients with incipient sepsis, FENa is far below the proposed cut-off of 1%, which might explain the poor performance of the FENa < 1% criterion. Unexpectedly, a persisting very low fractional urea excretion <31.5% was associated with intrinsic rather than transient AKI. A combined interpretation of high fractional sodium and urea excretion yielded a high negative predictive value for intrinsic AKI (92%) and persisting oliguria (95%), whereas a high fractional sodium and low fractional urea excretion had a 54% positive predictive value for patients remaining oliguric or developing intrinsic AKI.

Progress in the field of acute kidney injury has been hampered by lack of a uniform definition. In the last decade, several recommendations for a consensus definition have been proposed [13-15]. However, although all these definitions are based on both serum creatinine and urinary output criteria together, the latter parameter has been neglected in many studies. This is mainly because urinary output is mostly not available in administrative databases, or because people believe it is difficult to register urinary output. As a consequence, ongoing debate exists on the value of urinary output for defining AKI [18-23].

However, oliguria is also associated with hard outcomes, such as mortality [24], so it is now stressed that urinary output also should be taken into account when classifying AKI [15]. In our cohort, prevalence of the diagnosis of AKI decreased from 64.5% to 43% when urinary output was not taken into account. Whereas patients with transient AKI based on urinary output had similar levels of urinary NGAL production as did patients without AKI, patients with transient AKI based on creatinine had levels of urinary NGAL production intermediate between no AKI and intrinsic AKI (Figure 3), in line with the findings of Nejat et al. [8], where only the creatinine criterion was used. These results were confirmed when the fractional excretion of NGAL was used instead of urinary NGAL alone. These findings underline the concept that an increase in creatinine lags behind for early diagnosis of AKI, and that making a diagnosis based on urinary output might increase the potential for intervention at a stage when no tubular damage yet exists. Recently it was demonstrated in a *post hoc* analysis of the Chest study that fluid resuscitation with HES versus saline was more favorable when AKI was defined according to the diuresis criterion only, whereas saline was more favorable than HES when AKI was defined according to the creatinine criterion only [25]. In our cohort, the volume of HES administered was not different between no AKI and transient AKI and between transient AKI based on the diuresis criterion versus transient AKI based on the creatinine criterion.

Recently, it was demonstrated that assessment of urinary output can be easily and accurately achieved by registering it in 6- to 8-hour blocks [16]. This approach would make it possible even to monitor urinary output in patients without an indwelling bladder catheter, and maybe even outside the intensive care unit, at the hospital ward. This would be of importance, as it has been demonstrated that the majority of (fatal) cases of AKI develop unnoticed on the ward and are diagnosed with much delay [26].

There is discussion on the discriminatory value of fractional sodium excretion for the diagnosis of transient AKI. Usually, a cutoff of <1% is suggested as a diagnostic discriminant [27-29]. Our data indicate that this cutoff might be far too high, at least in sepsis, as 75% of the patients in our cohort had FENa <1%, and 50% even had a value <0.36%. Bagshaw *et al*. [30] recently reported an admission FENa below 1% in 57% of sepsis patients, being not different from nonsepsis patients. Darmon *et al*. [31] reported a median FENa on admission of 0.50%. Using RoC curve analysis, the same authors also found that a fractional excretion of 0.58% had the best discriminative power, resulting in a positive and negative predictive power for persistent AKI of 0.71 and 0.47, respectively [31]. In our cohort, we used the median, being 0.36%.

Our data highlight some other pitfalls for the interpretation of a high fractional sodium excretion. First, a high fractional excretion of sodium can also be a marker of tubular damage, due to active tubular secretion and reduced proximal tubular reabsorption. In our cohort, potentially in line with presumed tubular damage, we indeed found higher urinary NGAL levels in patients with a high versus low fractional sodium excretion, and this was most expressed in those who develop intrinsic AKI. Interestingly, in our cohort, patients with transient AKI had only higher urinary NGAL levels when they also had high fractional excretion of sodium >0.36% (Figure 4A), suggesting that some patients with transient AKI indeed develop some (minor) degree of tubular damage, whereas others do not. This also underpins that an increase in fractional sodium excretion is not always a positive sign, but can also be an indicator of subclinical tubular damage, which further adds to the low discriminative power of fractional sodium excretion, even when assessed in consecutive samples.

Second, a high fractional excretion of sodium might be caused by interference with use of diuretics. In our cohort, only a minority of patients were treated with diuretics, and those were equally distributed between AKI and no AKI. De Witte *et al.*[32] found much higher values of fractional sodium excretion in their cohort, but only a minority of their patients had sepsis, whereas 60% were treated with diuretics. Because of these two reasons, a high FENa can thus not be interpreted by itself.

Third, although the fractional excretion of sodium mainly decreases because of reduced urinary output and stimulation of sodium reabsorption, it can be speculated that fractional excretion of sodium also decreases when glomerular filtration decreases (for example, because of tubular obstruction). Therefore, although in our cohort, a very low fractional sodium excretion below <0.36% yielded a negative predictive value for intrinsic AKI of 87.3%, it is probably not a strong discriminative parameter, as was also suggested by Darmon *et al.*[31].

Surprisingly, and against our initial hypothesis, a low (<31.5%) fractional urea excretion at 4 hours was associated with intrinsic rather than with transient AKI, especially when it was persistent over time, whereas a value higher than 31.5% had a very high negative predictive value for intrinsic AKI. In patients with a fractional sodium excretion above 0.36%, the discriminatory value of fractional urea excretion was even more pronounced (Figure 4B).

Darmon *et al*. [31] reported an FEUrea of 39% in the no-AKI versus 41% in the transient AKI versus 32% in the intrinsic AKI group, and concluded that FEUrea had a low value for discriminating intrinsic AKI. They based their analysis on admission FEUrea, whereas we used the value 4 hours after admission, when some attempts to restore low glomerular filtration pressure (for example, by a fluid challenge or start of vasopressors) have already been installed. However, data based on FEUrea and FENa at admission showed comparable results, although less impressive (data not shown). Our findings are in line with those of De Witte *et al*., who also found a reasonable value of FEurea <40% as a parameter to discriminate transient from intrinsic AKI [32]. As a low fractional excretion of urea apparently represents a low glomerular filtration, we hypothesized that a combination of fractional sodium and urea excretion would yield the optimal prediction of patients with a substantial chance to respond positively to attempts to restore glomerular filtration. Indeed, in our cohort, a combination of a high fractional sodium and urea excretion yielded a 95% probability of restoring diuresis, and the AKI being transient rather than intrinsic, whereas a persistently low fractional urea excretion in combination with a high fractional sodium excretion was suggestive for intrinsic AKI.

Debate exists on the discriminatory value of NGAL to predict or diagnose AKI in clinical conditions, as much overlap in NGAL levels is seen between patients with versus without AKI [33]. NGAL is a 25-kDa molecule that is filtered into the primary urine at the glomerular level. In sepsis, serum levels of NGAL increase exponentially [34,35], even in the absence of AKI [36], which can result in increased urinary levels as well [37]. Once filtered, NGAL is reabsorbed by the tubular epithelium through the megalin receptor, and this through competitive binding [38].

Although a recent study indicated that urinary NGAL stems from local production in the thick ascending limb of the loop of Henle when stress factors are applied, evidence suggests that urinary NGAL can derive from the systemic circulation [37,39]. This indicates that the presence of NGAL in the urine in sepsis patients cannot automatically be considered as a marker of tubular damage *per se*. In addition, some patients with transient AKI might have some degree of tubular damage, which might explain the relatively low value of NGAL to discriminate transient from intrinsic AKI in this cohort of sepsis patients.

A strength of this study is that it describes one of the largest cohorts of sepsis patients in detail for different patho-physiologic aspects of AKI, such as urinary and serum biomarkers, and urinary output. A limitation on the other hand is that it is observational and hence no causal assumptions can be made.

## Conclusion

Urinary output is an early and sensitive marker of AKI, which might incite intervention before tubular damage has occurred. Low fractional excretion of sodium (<1%) and urea (<35%) is very frequent in sepsis patients, and it might be necessary to define lower discriminatory cut-off values as an indication of maximal tubular reabsorption and thus intact tubular function as the ones used at present. A combination of high fractional excretion of sodium and urea has a high negative predictive value for intrinsic AKI, whereas a high fractional sodium excretion and a low fractional urea excretion are associated with intrinsic AKI in 54% of cases.

## Key messages

 ● A low FENa and FEUrea is highly prevalent in the first hours of sepsis.

 ● Neither FENa, FEUrea, nor NGAL alone has sufficient power to discriminate intrinsic from transient AKI.

 ● The combination of a high FENa and a low FEUrea is associated with intrinsic AKI, whereas a high FENa and a high FEUrea are predictive of transient AKI.

 ● Omitting the urinary output criterion leads to underdiagnosis of AKI.

 ● Oliguria is an earlier sign of impending AKI than increase in serum creatinine.

## Abbreviations

AKI: Acute kidney injury; AKIboth: AKI based on both the urinary output and the serum creatinine criterion; AKIc: AKI based on the serum creatinine criterion; AKIuo: AKI based on the urinary output criterion; AUC ROC: Area under a receiver operating characteristic curve; d0t0: Time point at admission; d0t4: Time point 4 hours after admission; d1: Time point 24 hours after admission; FENa: Fractional excretion of sodium; FEUrea: Fractional excretion of urea; LFABP: Liver fatty acid-binding protein; NGAL: Neutrophile gelatinase-associated lipocalin; RIFLE: Risk injury, failure, loss of kidney function, end-stage renal disease; TAKI: Transient acute kidney injury.

## Competing interests

The authors have no financial or nonfinancial competing interests to declare.

## Authors’ contributions

JV coordinated the study, collected the samples, and acquired all necessary information for analysis and interpretation of the data and writing of the draft. GG was responsible for all laboratory analyses and revised the manuscript critically. EH helped including patients and revised the manuscript critically. AD helped including patients and revised the manuscript critically. RV helped designing the study and revised the manuscript critically. RV also participated in carrying out the statistical analysis. WVB conceived of the study and participated in its design and coordination and helped to draft the manuscript. WVB also performed statistical analysis. All authors read and approved the final manuscript.

## References

[B1] Bellomo R Bagshaw S Langenberg C Ronco C Pre-renal azotemia: a flawed paradigm in critically ill septic patients? Contrib Nephrol 2007 17 1 9 17464109 10.1159/000102008

[B2] Bellomo R Wan L Langenberg C May C Septic acute kidney injury: new concepts Nephron Exp Nephrol 2008 17 e95 100 10.1159/000142933 18802375

[B3] Schrier RW Wang W Acute Renal Failure and Sepsis N Engl J Med 2004 17 159 169 10.1056/NEJMra032401 15247356

[B4] Prowle JR Ishikawa K May CN Bellomo R Renal blood flow during acute renal failure in man Blood Purif 2009 17 216 225 10.1159/000230813 19648741

[B5] Prowle JR Molan MP Hornsey E Bellomo R Measurement of renal blood flow by phase-contrast magnetic resonance imaging during septic acute kidney injury: a pilot investigation Critical.Care Medicine 2012 17 6 1768 76 10.1097/CCM.0b013e318246bd85 22487999

[B6] Wan L Bagshaw SM Langenberg C Saotome T May C Bellomo R Pathophysiology of septic acute kidney injury: what do we really know? Crit Care Med 2008 17 S198 S203 10.1097/CCM.0b013e318168ccd5 18382194

[B7] Langenberg C Wan L Egi M May CN Bellomo R Renal blood flow in experimental septic acute renal failure Kidney Int 2006 17 1996 2002 10.1038/sj.ki.5000440 16641923

[B8] Nejat M Pickering JW Devarajan P Bonventre JV Edelstein CL Walker RJ Endre ZH Some biomarkers of acute kidney injury are increased in pre-renal acute injury Kidney Int 2012 17 1254 1262 10.1038/ki.2012.23 22418979 PMC3365288

[B9] Doi K Katagiri D Negishi K Hasegawa S Hamasaki Y Fujita T Matsubara T Ishii T Yahagi N Sugaya T Noiri E Mild elevation of urinary biomarkers in prerenal acute kidney injury Kidney Int 2012 17 1114 1120 10.1038/ki.2012.266 22854644

[B10] Bouchard J Soroko SB Chertow GM Himmelfarb J Ikizler TA Paganini EP Mehta RL Fluid accumulation, survival and recovery of kidney function in critically ill patients with acute kidney injury Kidney Int 2009 17 422 427 10.1038/ki.2009.159 19436332

[B11] Schrier RW Fluid administration in critically ill patients with acute kidney injury Clin J Am Soc Nephrol 2010 17 733 739 10.2215/CJN.00060110 20167687

[B12] Kidney disease: Improving Global Outcomes (KDIGO) Acute Kidney Injury Work Group. KDIGO Clinical Practice Guideline for Acute Kidney Injury Kidney Int.Suppl 2012 17 1 138

[B13] Bellomo R Ronco C Kellum JA Mehta RL Palevsky P Acute renal failure - definition, outcome measures, animal models, fluid therapy and information technology needs: the Second International Consensus Conference of the Acute Dialysis Quality Initiative (ADQI) Group Crit Care 2004 17 R204 R212 10.1186/cc2872 15312219 PMC522841

[B14] Mehta RL Kellum JA Shah SV Molitoris BA Ronco C Warnock DG Levin A Acute Kidney Injury Network: report of an initiative to improve outcomes in acute kidney injury Crit Care 2007 17 R31 10.1186/cc5713 17331245 PMC2206446

[B15] Fliser D Laville M Covic A Fouque D Vanholder R Juillard L Van BW A European Renal Best Practice (ERBP) position statement on the Kidney Disease Improving Global Outcomes (KDIGO) clinical practice guidelines on acute kidney injury: part 1: definitions, conservative management and contrast-induced nephropathy Nephrol Dial Transplant 2012 17 4263 4272 23045432 10.1093/ndt/gfs375 PMC3520085

[B16] Macedo E Malhotra R Claure-Del GR Fedullo P Mehta RL Defining urine output criterion for acute kidney injury in critically ill patients Nephrol Dial Transplant 2011 17 509 515 10.1093/ndt/gfq332 20562094 PMC3108356

[B17] American College of Chest Physicians/Society of Critical Care Medicine Consensus Conference: definitions for sepsis and organ failure and guidelines for the use of innovative therapies in sepsis Crit Care Med 1992 17 864 874 10.1097/00003246-199206000-00025 1597042

[B18] Bivet FG Nonuse of RIFLE classification urine output criteria: biases for acute kidney injury biomarkers performance assessment? Crit Care Med 2012 17 1692 1693 22511170 10.1097/CCM.0b013e318246b72a

[B19] Cruz DN Bolgan I Perazella MA Bonello M de CM Corradi V Polanco N Ocampo C Nalesso F Piccinni P Ronco C **North East Italian Prospective Hospital Renal Outcome Survey on Acute Kidney Injury (NEiPHROS-AKI): targeting the problem with the RIFLE Criteria** Clin J Am Soc Nephrol 2007 17 418 425 10.2215/CJN.03361006 17699446

[B20] Joannidis M Classification of acute kidney injury: are we there yet? Intensive Care Med 2007 17 572 574 10.1007/s00134-007-0536-z 17310364

[B21] Mandelbaum T Scott DJ Lee J Mark RG Malhotra A Waikar SS Howell MD Talmor D Outcome of critically ill patients with acute kidney injury using the Acute Kidney Injury Network criteria Crit Care Med 2011 17 2659 2664 21765352 10.1097/CCM.0b013e3182281f1b PMC3213281

[B22] Prowle JR Liu YL Licari E Bagshaw SM Egi M Haase M Haase-Fielitz A Kellum JA Cruz D Ronco C Tsutsui K Uchino S Bellomo R Oliguria as predictive biomarker of acute kidney injury in critically ill patients Crit Care 2011 17 R172 10.1186/cc10318 21771324 PMC3387614

[B23] Ricci Z Cruz D Ronco C The RIFLE criteria and mortality in acute kidney injury: A systematic review Kidney Int 2008 17 538 546 10.1038/sj.ki.5002743 18160961

[B24] Macedo E Malhotra R Bouchard J Wynn SK Mehta RL Oliguria is an early predictor of higher mortality in critically ill patients Kidney Int 2011 17 760 767 10.1038/ki.2011.150 21716258

[B25] Myburgh JA Finfer S Bellomo R Billot L Cass A Gattas D Glass P Lipman J Liu B McArthur C McGuinness S Rajbhandari D Taylor CB Webb SA Hydroxyethyl starch or saline for fluid resuscitation in intensive care N Engl J Med 2012 17 1901 1911 10.1056/NEJMoa1209759 23075127

[B26] MacLeod A NCEPOD report on acute kidney injury-must do better Lancet 2009 17 1405 1406 10.1016/S0140-6736(09)61843-2 19854359

[B27] Bagshaw SM Langenberg C Bellomo R Urinary biochemistry and microscopy in septic acute renal failure: a systematic review Am J Kidney Dis 2006 17 695 705 10.1053/j.ajkd.2006.07.017 17059988

[B28] Carvounis CP Nisar S Guro-Razuman S Significance of the fractional excretion of urea in the differential diagnosis of acute renal failure Kidney Int 2002 17 2223 2229 10.1046/j.1523-1755.2002.00683.x 12427149

[B29] Pepin MN Bouchard J Legault L Ethier J Diagnostic performance of fractional excretion of urea and fractional excretion of sodium in the evaluations of patients with acute kidney injury with or without diuretic treatment Am J Kidney Dis 2007 17 566 573 10.1053/j.ajkd.2007.07.001 17900456

[B30] Bagshaw SM Haase M Haase-Fielitz A Bennett M Devarajan P Bellomo R A prospective evaluation of urine microscopy in septic and non-septic acute kidney injury Nephrol Dial Transplant 2012 17 582 588 10.1093/ndt/gfr331 21669886

[B31] Darmon M Vincent F Dellamonica J Schortgen F Gonzalez F Das V Zeni F Brochard L Bernardin G Cohen Y Schlemmer B Diagnostic performance of fractional excretion of urea in the evaluation of critically ill patients with acute kidney injury: a multicenter cohort study Crit Care 2011 17 R178 10.1186/cc10327 21794161 PMC3387621

[B32] Dewitte A Biais M Petit L Cochard JF Hilbert G Combe C Sztark F Fractional excretion of urea as a diagnostic index in acute kidney injury in intensive care patients J Crit Care 2012 17 505 510 10.1016/j.jcrc.2012.02.018 22520491

[B33] Vanmassenhove J Vanholder R Nagler E Van BW Urinary and serum biomarkers for the diagnosis of acute kidney injury: an in-depth review of the literature Nephrol Dial Transplant 2013 17 254 273 10.1093/ndt/gfs380 23115326

[B34] Bagshaw SM Bennett M Haase M Haase-Fielitz A Egi M Morimatsu H D'Amico G Goldsmith D Devarajan P Bellomo R Plasma and urine neutrophil gelatinase-associated lipocalin in septic versus non-septic acute kidney injury in critical illness Intensive Care Med 2010 17 452 461 10.1007/s00134-009-1724-9 19956924

[B35] Wheeler DS Devarajan P Ma Q Harmon K Monaco M Cvijanovich N Wong HR Serum neutrophil gelatinase-associated lipocalin (NGAL) as a marker of acute kidney injury in critically ill children with septic shock Crit Care Med 2008 17 1297 1303 10.1097/CCM.0b013e318169245a 18379258 PMC2757115

[B36] Martensson J Bell M Oldner A Xu S Venge P Martling CR Neutrophil gelatinase-associated lipocalin in adult septic patients with and without acute kidney injury Intensive Care Med 2010 17 1333 1340 10.1007/s00134-010-1887-4 20397003

[B37] Pedersen SS Keller AK Rehling M Birn H Jespersen B NGAL excretion is higher from the healthy side than from the injured side in unilateral acute kidney injury Scand J Clin Lab Invest 2012 17 510 512 10.3109/00365513.2012.692809 22671281

[B38] Hvidberg V Jacobsen C Strong RK Cowland JB Moestrup SK Borregaard N The endocytic receptor megalin binds the iron transporting neutrophil-gelatinase-associated lipocalin with high affinity and mediates its cellular uptake FEBS Lett 2005 17 773 777 10.1016/j.febslet.2004.12.031 15670845

[B39] Paragas N Qiu A Zhang Q Samstein B Deng SX Schmidt-Ott KM Viltard M Yu W Forster CS Gong G Liu Y Kulkarni R Mori K Kalandadze A Ratner AJ Devarajan P Landry DW D'Agati V Lin CS Barasch J The Ngal reporter mouse detects the response of the kidney to injury in real time Nat Med 2011 17 216 222 10.1038/nm.2290 21240264 PMC3059503

